# Pin1 and JNK1 cooperatively modulate TAp63γ

**DOI:** 10.1002/2211-5463.13109

**Published:** 2021-02-19

**Authors:** Xueying Fan, Wei He, Ke Hu, Huimin Chen, Li Chen, Shijie Fan, Chenghua Li

**Affiliations:** ^1^ Center of Growth, Metabolism and Aging Key Laboratory of Biological Resources and Ecological Environment of Ministry of Education College of Life Sciences, Sichuan University Chengdu China; ^2^ Department of Hepato‐Pancreato‐Biliary Surgery 363 Hospital Chengdu China

**Keywords:** JNK1, Pin1, TAp63γ, transactivity

## Abstract

The *p63* gene encodes at least 10 isoforms, which can be classified into TA and ∆N isotypes (TAp63 and ∆Np63 proteins) according to their differences at the N termini. TAp63γ is an important transcription factor. We previously reported that peptidyl‐prolyl isomerase (PPI) Pin1 directly binds to TAp63γ protein and identified that serine 12 (S_12_) in the transactivation domain (TAD) of TAp63γ is required for regulation of its transcriptional activity. In the present study, we report that Pin1 stimulates transcriptional and pro‐apoptotic activities of TAp63γ; this Pin1‐mediated stimulation may depend on phosphorylation of S_12_ mediated by JNK1 and results in striking activation of TAp63γ. JNK1 represses transactivity of TAp63γ in cells without abundant Pin1 proteins and enhances it in the presence of sufficient levels of Pin1. Collectively, our data suggest a novel mechanism for regulation of TAp63γ transactivity: TAp63γ with unphosphorylated S_12_ is moderately active, phosphorylation at this residue (pS_12_) makes it hypoactive, and Pin1 binds to the pS_12_‐P_13_ motif and makes TAp63γ hyperactive. Our findings will aid in the elucidation of the mechanism underlying modulation of TAp63γ.

## Graphical abstract

When the serine 12 residue (S_12_) in the transactivation domain of TAp63γ is unphosphorylated, TAp63γ is moderately active to transactivate its downstream genes. Phosphorylation at this residue (pS_12_) can be mediated by kinase JNK1, leading to a repression of TAp63γ transactivity; isomerization of the pS_12_‐P_13_ motif can be mediated by Pin1, which makes TAp63γ hyperactive.

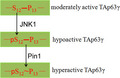

**Abbreviations**
A: alanine
CL‐PARP1: cleaved PARP1
CoIP: co‐immunoprecipitation
IB: immunoblotting
IP: immunoprecipitation
MTT: 3‐(4,5‐dimethylthiazol‐2‐yl)‐2,5‐diphenyl‐tetrazolium bromide
P: proline
PPI: peptidyl‐prolyl isomerase
pS: phosphorylated serine
S: serine
SAM: sterile alpha motif
T: threonine
TAD: transactivation domain
TID: transinhibition domain
Y: tyrosine

The *p63* gene belongs to the *p53* family and encodes at least 10 isoforms, which can be classified into TA and ∆N isotypes (TAp63 and ∆Np63 proteins) according to their differences at the N termini. TAp63s contain the full transactivation domain (TAD) at the N termini, while ∆Np63 isotypes have an incomplete TAD with a weaker transactivity. After transcription, both TA and ∆N isotypes can be spliced into mRNAs with different 3’ termini, generating at least 5 different C termini, α, β, γ, δ, and ε. Among them, the γ types miss the sterile alpha motif (SAM) and the transinhibition domain (TID) at their C termini compared with the α isoform of p63 proteins [1, 2, 3]. TAp63 proteins express at relatively lower levels in somatic cells. However, like p53, these TA isoforms of p63 play key roles in cell cycle arrest and apoptotic cell death via transactivating pro‐apoptotic factors such as p21, Puma, Bax, and Noxa [4, 5, 6]. Thus, TAp63s function as quality control factors in the female germline upon genotoxic stress [7, 8, 9, 10]. Studies with mouse models demonstrate that specific knockout of TAp63 can cause premature aging [11, 12] and metabolic syndrome [13]. These TAp63‐null mice are also highly tumor prone and develop metastatic diseases [11, 14], reaffirming the tumor suppressor functions of TAp63 proteins. Data from Ernesto Bruno group suggest that TAp63 suppresses recurrence of nasal polyps [15]. According to reports from group of Esther H. Chang, miR‐130b and TAp63 form a feed‐forward loop, and this miR‐130b/TAp63 axis is a druggable pathway that has the potential to uncover broad‐spectrum therapeutic options for the majority of p53‐altered cancers [16]. It has been reported that TAp63 may also function as a repressor of transcription [17]. Recently, Suenaga Y and Nakagawara A et al found that TAp63 restrains neuroblastoma growth via repressing MYCN/NCYM bidirectional transcription [18]. As a short isoform of TAp63, TAp63γ is assumed to have a high activity to mediate transcription and apoptosis, since it lacks TID and SAM at the C terminus [1]. Some recent reports demonstrate that TAp63γ promotes myogenic differentiation, osteoblastic differentiation, and cartilage development [19, 20, 21].

Due to their key roles in cell cycle control, both expression levels and activities of p63 proteins are tightly regulated in cells [2]. According to data from our group and other laboratories, p63 proteins undergo various post‐translational modifications including phosphorylation, ubiquitination, and isomerization [2, 22, 23, 24, 25, 26, 27, 28]. Particularly, we previously reported that peptidyl‐prolyl isomerase (PPI) Pin1 physically interacts with several protein isoforms of p63, including TAp63α, ∆Np63α, and TAp63γ; Pin1 specifically binds to the T‐P‐P‐P‐P‐Y motif in the SAM of p63α proteins and inhibits the proteasomal degradation of them [22]. However, γ isoforms lack the T‐P‐P‐P‐P‐Y motif and SAM. Therefore, the binding sites and effects of Pin1 on TAp63γ remain obscure. In another study, we found that c‐Jun N‐terminal kinase 1 (JNK1) may phosphorylate TAp63γ at serine 12 and impair its transactivity and pro‐apoptotic activity [27]. In the present work, we find that Pin1 stimulates transcriptional and pro‐apoptotic activities of TAp63γ; S12A mutation in TAp63γ impairs its physical interaction with Pin1 and deprives Pin1‐mediated stimulation of TAp63γ; we further find that Pin1 strikingly reverses JNK1‐repressed transactivity of TAp63γ and makes it hyperactive. Our findings are helpful to elucidate how transactivity of TAp63γ is modulated.

## Materials and methods

### Cell culture, transfection, and plasmids

Saos‐2, Hela, and H1299 cells were cultured in Modified McCoy's 5a Medium (BI) supplemented with 10% FBS (BI) and 1% penicillin G/streptomycin (Hyclone, Logan, UT, USA) at 37 °C in a humidified 5% CO_2_ incubator. Transient transfection was performed with Entranster™‐H4000 (Engreen Biosystem, Beijing, China), and total amounts of plasmid DNA were balanced with corresponding vectors for each transfection. Constructs of pcDNA3.1‐HA‐TAp63γ, pcDNA3.1‐HA‐TAp63γ(S12A), pcDNA3.1‐Pin1, pcDNA3.1‐Pin1(W34A), and pcDNA3.1‐JNK1 were previously described [22, 27, 29]. JNK1 siRNA and scrambled control were purchased from Santa Cruz Biotechnology (Dallas, TX, USA). Cell viabilities were determined by 3‐(4,5‐dimethylthiazol‐2‐yl)‐2,5‐diphenyl‐tetrazolium bromide (MTT; Promega, Madison, WI, USA) as described in the instruction.

### Immunoprecipitation and immunoblotting analysis

Immunoprecipitation (IP) and immunoblotting (IB) analyses were performed as previously described [22, 27]. Antibodies used were specific for Pin1 (rabbit polyclonal antibody; Cell Signaling Technology, Beverly, MA, USA; 1 : 1000), JNK1 (rabbit polyclonal antibody; Abcam, Cambridge, MA, USA; 1 : 1000), HA (mouse monoclonal antibody; Millipore, Billerica, CA, USA;1 : 500), p63 (rabbit polyclonal antibody; Zen‐bio, Chengdu, Sichuan, China; 1 : 1000), PARP1 (rabbit polyclonal antibody; Zen‐bio, Chengdu, Sichuan, China; 1 : 2000), and GAPDH (rabbit polyclonal antibody; Zen‐bio, Chengdu, Sichuan, China; 1 : 1000). Blots were detected using an ECL system (GE Amersham Pharmacia Biotech, Boston, MA, USA).

### Luciferase reporter assay

Luciferase assays were performed as described previously [22, 27]. Saos‐2 cells were transfected with a mixture of Bax‐Luc and pRL‐TK‐Renilla plus indicated plasmids or siRNAs. Total amount of DNAs or RNAs was balanced with control vectors or scramble control RNAs. Cells were harvested at 48 h post‐transfection and lysed in Passive Lysis Buffer (Promega). Lysates were analyzed for firefly and Renilla luciferase activities using the Dual Luciferase Reagent Assay Kit (Promega). Luminescence was measured in a luminometer. Relative luciferase activity was determined by normalizing luciferase activity with Renilla.

### Statistical analysis

All experiments were carried out in triplicate. Two‐tailed *t‐*test was used for comparison between two groups. *P* < 0.05 was considered statistically significant. All the error bars indicate SD.

## Results

### Pin1 enhances TAp63γ‐induced transcription and apoptosis

**Fig. 1:**
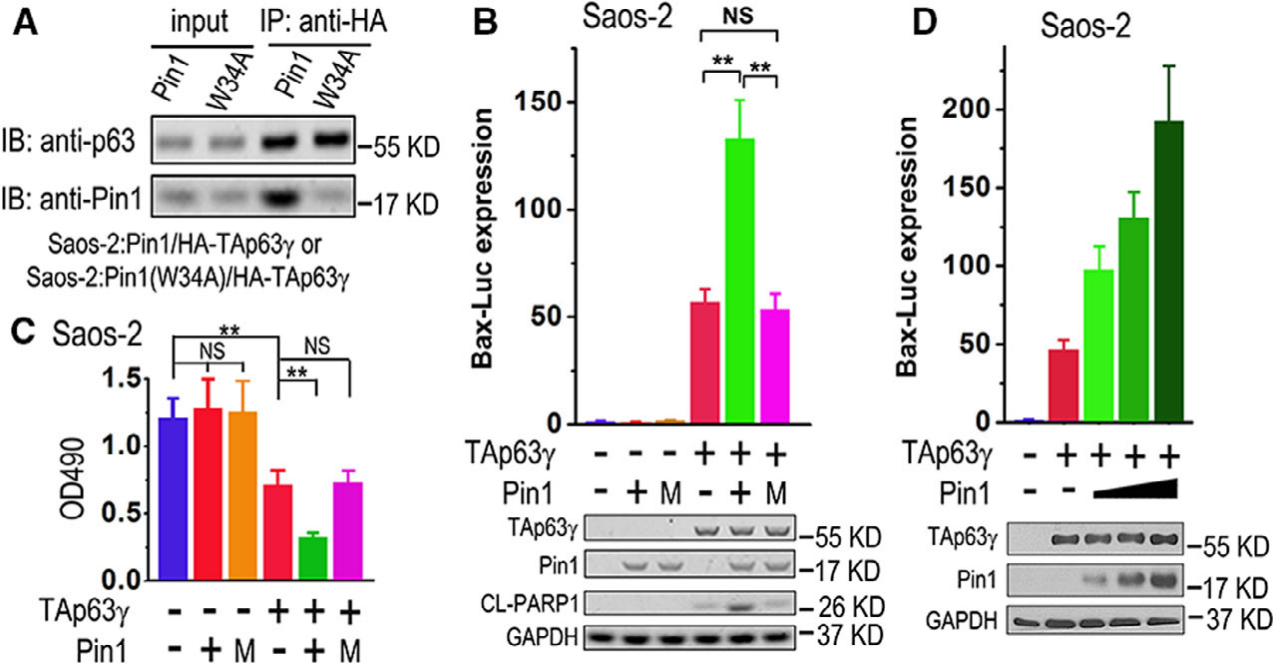
Pin1 enhances TAp63γ‐induced transcription and apoptosis. (A) Saos‐2 cells transfected with HA‐TAp63γ, plus Pin1 or its W34A mutant, were lysed and subjected to IP with anti‐HA. The cell lysates (inputs) or IP products were subjected to immunoblot (IB) analysis with indicated primary antibodies. (B) Saos‐2 cells were transfected with a mixture of Bax‐Luc and TK‐Renilla plus indicated plasmids. M, W34A mutant Pin1. Firefly and Renilla luciferase activities were measured, while IB analyses were performed to detect indicated proteins. The Bax‐Luc activity was normalized to Renilla activity and presented as Bax‐Luc expression level with SD (*n* = 3). Bax‐Luc expression in cells transfected with Bax‐Luc/TK‐Renilla mixture alone was set as 1. Two‐tailed *t‐*test was used for comparison between two groups; **P < 0.01; NS, nonsignificant. (C) Saos‐2 cells transfected with indicated plasmids were subjected to cell survival measurement with 3‐(4,5‐dimethylthiazol‐2‐yl)‐2,5‐diphenyl‐tetrazolium bromide (MTT). Cell viabilities were presented as optical density values at the wavelength of 490 nm (OD490) with SD (*n* = 3). Two‐tailed *t‐*test was used for comparison between two groups; **P < 0.01; NS, nonsignificant. (D) Saos‐2 cells were transfected with a mixture of Bax‐Luc and TK‐Renilla plus HA‐TAp63γ and increasing amounts of Pin1 plasmid as indicated. Bax‐Luc expression levels were measured and presented as mentioned above, while IB analyses were performed to detect indicated proteins. The error bars indicate SD (*n* = 3).

In a previous study, we performed a pull‐down experiment and found that TAp63γ protein forms a complex with PPI Pin1; mutation on tryptophan 34 to alanine (W34A) in Pin1, which was reported to disrupt the binding of this isomerase to its substrates, significantly impairs its physical interaction with TAp63γ [22]. To confirm this interaction in mammalian cells, we transiently overexpressed HA‐tagged TAp63γ (HA‐TAp63γ), along with wild‐type Pin1 or its W34A mutant, in human osteosarcoma cell Saos‐2, and performed a co‐immunoprecipitation (CoIP) assay. The results demonstrate that Pin1 can form a stable complex with TAp63γ, while W34A mutation in Pin1 significantly impairs this interaction (Fig. 1A). Bax is a downstream gene of TAp63; luciferase reporter driven by Bax promoter (Bax‐Luc) can be used to measure the transactivity of TAp63 proteins [22]. To further investigate whether Pin1 modulates transactivity of TAp63γ, we performed a luciferase reporter assay. The results demonstrate that the wild‐type Pin1, but not its W34A mutant (M), significantly enhances TAp63γ‐mediated expression of Bax‐Luc (Fig. 1B). On the other hand, we used MBC1‐4‐Luc reporter as a nonresponsive promoter control and found that neither TAp63γ nor Pin1 can activate its expression (data not shown) [30], indicating the specific regulation of both proteins on Bax‐Luc expression. The IB analysis results reveal that neither wild‐type Pin1 nor its W34A M affects the expression level of TAp63γ; wild‐type Pin1, but not the mutant, significantly increases the level of cleaved PARP1 (CL‐PARP1), which is a molecular marker of cell apoptosis and can be induced by TAp63γ (Fig. 1B). These effects of Pin1 and TAp63γ are consistent with the results of cell survival/proliferation assay: wild‐type Pin1, but not its W34A mutant, significantly aggravates cell proliferation/survival inhibition of TAp63γ (Fig. 1C). Further, we found that Pin1 stimulates TAp63γ‐mediated expression of Bax‐Luc in a dose‐dependent manner (Fig. 1D). These results suggest that Pin1 stimulates transcriptional and pro‐apoptotic activities of TAp63γ.

### Serine 12 in the transactivation domain of TAp63γ is crucial to Pin1‐mediated stimulation

**Fig. 2:**
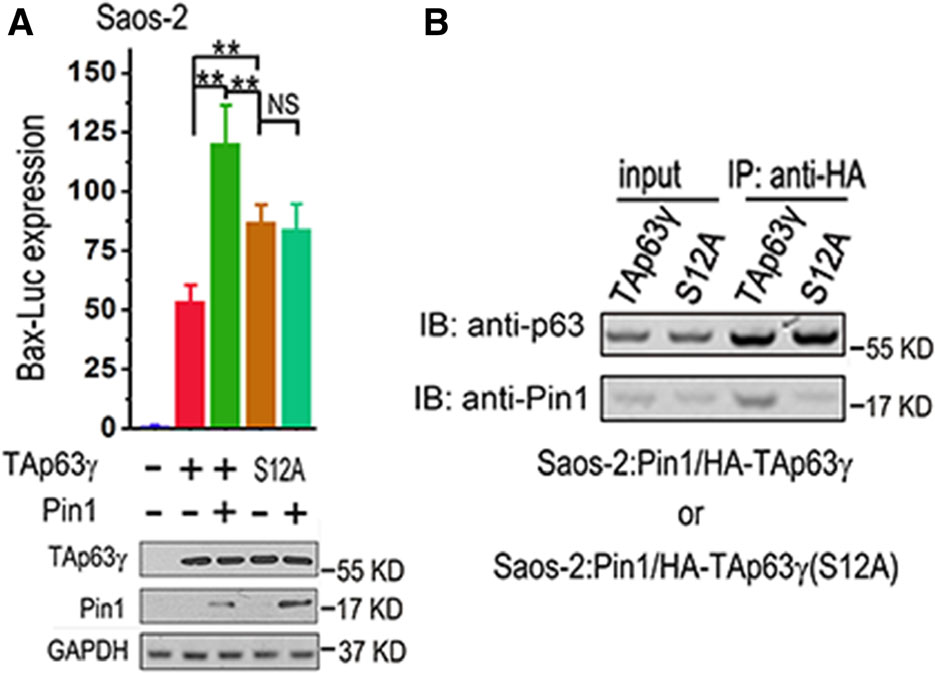
Serine 12 in the TAD of TAp63γ is crucial to Pin1‐mediated stimulation. (A) Saos‐2 cells were transfected with a mixture of Bax‐Luc and TK‐Renilla plus indicated plasmids. S12A, S12A mutant TAp63γ. Bax‐Luc expression levels were measured and presented as mentioned above (*n* = 3), while IB analyses were performed to detect indicated proteins. The error bars indicate SD. Two‐tailed *t‐*test was used for comparison between two groups; **P < 0.01; NS, nonsignificant. (B) Saos‐2 cells transfected with HA‐TAp63γ, plus Pin1 or its W34A mutant, were lysed and subjected to IP with anti‐HA. The cell lysates (inputs) or IP products were subjected to IB analysis with indicated primary antibodies.

In another previous report from our group, we found that serine 12 (S_12_) is crucial to transactivity of TAp63γ [27]. S_12_ is followed by a proline residue (P_13_), composing a putative Pin1 modification site [22]. It is well known that phosphorylation of the serine or threonine followed by proline is essential for the binding of Pin1 [31]. As a PPI, Pin1 mediates isomerization of proline, which is prevented by phosphorylation of the adjacent serine or threonine residue (pS‐P or pT‐P) [32]. This isomerization offers a molecular switch for recruitment of protein binding or post‐translational modification and modulates transactivity of multiple transcription factors [33, 34, 35]. To investigate whether this pS_12_‐P_13_ site is involved in Pin1‐mediated stimulation of TAp63γ (Fig. 1B,D), we tested the effect of Pin1 on expression of Bax‐Luc mediated by S12A mutant TAp63γ, which loses phosphorylation at this site. The results demonstrate that though S12A mutation enhances transactivity of TAp63γ, the expression of Bax‐Luc mediated by the mutant cannot be stimulated by Pin1 (Fig. 2A). The results of CoIP show that TAp63γ readily binds to Pin1 and this physical interaction can be significantly impaired by S12A mutation (Fig. 2B). These results reveal that serine 12 in the TAD of TAp63γ is crucial to its interaction with Pin1 and Pin1‐mediated stimulation.

### Pin1 strikingly reverses JNK1‐repressed transcriptional and pro‐apoptotic activities of TAp63γ and makes it hyperactive

**Fig. 3:**
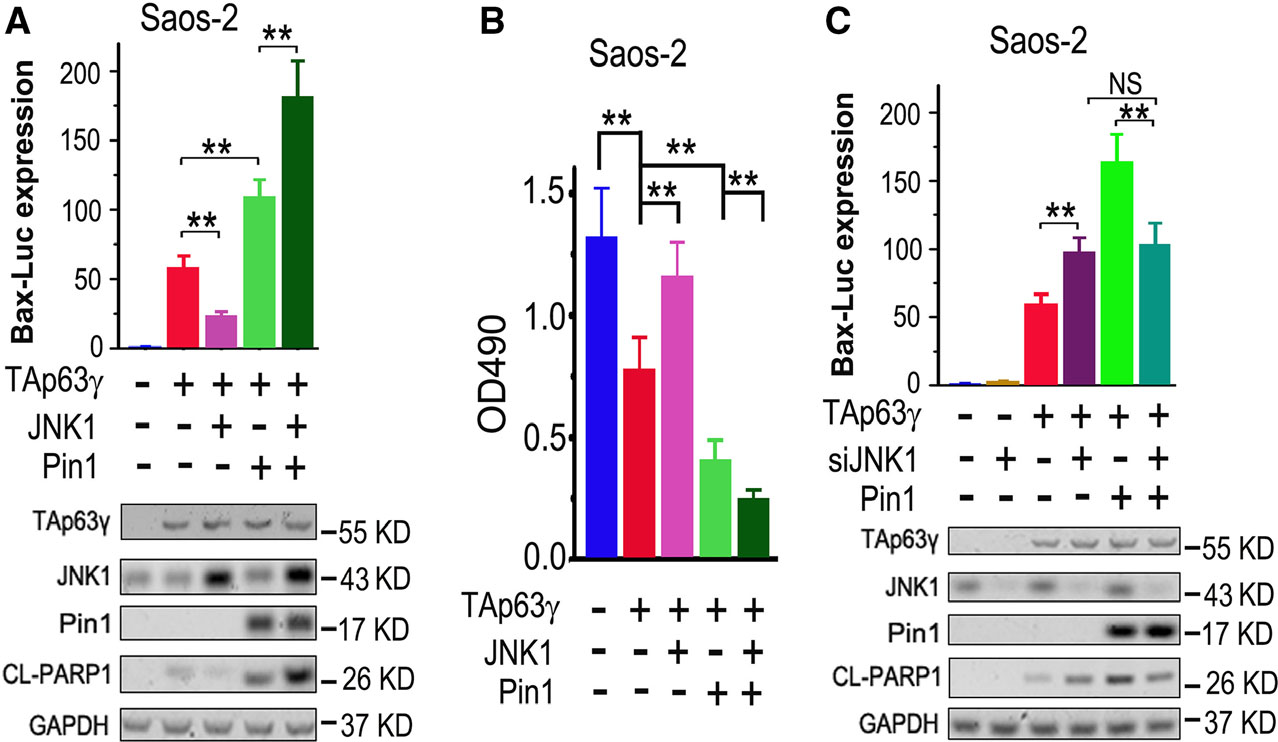
Pin1 strikingly reverses JNK1‐repressed activity of TAp63γ and makes it hyperactive. (A) Saos‐2 cells were transfected with a mixture of Bax‐Luc and TK‐Renilla plus indicated plasmids. Bax‐Luc expression levels were measured and presented as mentioned above (*n* = 3), while IB analyses were performed to detect indicated proteins. Two‐tailed *t‐*test was used for comparison between two groups; **P < 0.01. (B) Saos‐2 cells transfected with indicated plasmids were subjected to cell survival measurement with MTT. Cell viabilities were presented as mentioned above (*n* = 3). Two‐tailed *t‐*test was used for comparison between two groups; **P < 0.01. (C) Saos‐2 cells were transfected with a mixture of Bax‐Luc and TK‐Renilla plus indicated plasmids or siRNAs. Bax‐Luc expression levels were measured and presented as mentioned above, while IB analyses were performed to detect indicated proteins. Two‐tailed *t‐*test was used for comparison between two groups; **P < 0.01; NS, nonsignificant. The error bars (A–C) indicate SD (*n* = 3).

In our previous report mentioned above, we found that JNK1 can phosphorylate TAp63γ at serine 12, resulting in a repression of its transcriptional and pro‐apoptotic activities [27]. To further investigate the effects of JNK1 and Pin1 on TAp63γ, we transfected JNK1 and (or) Pin1 along with TAp63γ into Saos‐2 cells. The results of luciferase reporter assay show that TAp63γ‐mediated Bax‐luc expression is repressed by JNK1 but boosted by Pin1; unexpectedly, simultaneous overexpression of JNK1 can further enhance Pin1‐mediated activation of TAp63γ (Fig. 3A). The IB analysis reveals that overexpression of JNK1 or Pin1 has no significant effects on the protein level of TAp63γ; JNK1 significantly impairs the production of CL‐PARP1 induced by TAp63γ; on the contrary, Pin1 obviously promotes TAp63γ‐induced CL‐PARP1; intriguingly, simultaneous overexpression of Pin1 and JNK1 can strikingly exacerbate cleavage of PARP1 induced by TAp63γ (Fig. 3A). In line with the PARP1 cleavage results, TAp63γ‐induced inhibition of cell survival/proliferation is rescued by JNK1 and intensified by Pin1, while further exacerbated by simultaneous overexpression of Pin1 and JNK1 (Fig. 3B). Next, we knocked down endogenous JNK1 with siRNA used previously [27] and tested the Pin1‐mediated activation of TAp63γ. The results of IB analysis show that the specific siRNA can effectively ablate endogenous JNK1 in Saos‐2 cells; TAp63γ induces the production of CL‐PARP1, which can be further increased by the ablation of JNK1; overexpression of both TAp63γ and Pin1 makes an even higher CL‐PARP1 level, while simultaneous knockdown of JNK1 impairs the effect of Pin1 on TAp63γ‐induced production of CL‐PARP1 (Fig. 3C). The luciferase reporter assay demonstrates that ablation of JNK1 significantly increases TAp63γ‐mediated expression of Bax‐Luc; ablation of JNK1 abrogates the effect of Pin1 on TAp63γ‐mediated expression of Bax‐Luc (Fig. 3C). These results suggest that Pin1 strikingly reverses JNK1‐repressed transcriptional and pro‐apoptotic activities of TAp63γ and makes it hyperactive.

### JNK1 may repress or promote transactivity of TAp63γ depending on Pin1 level

**Fig. 4:**
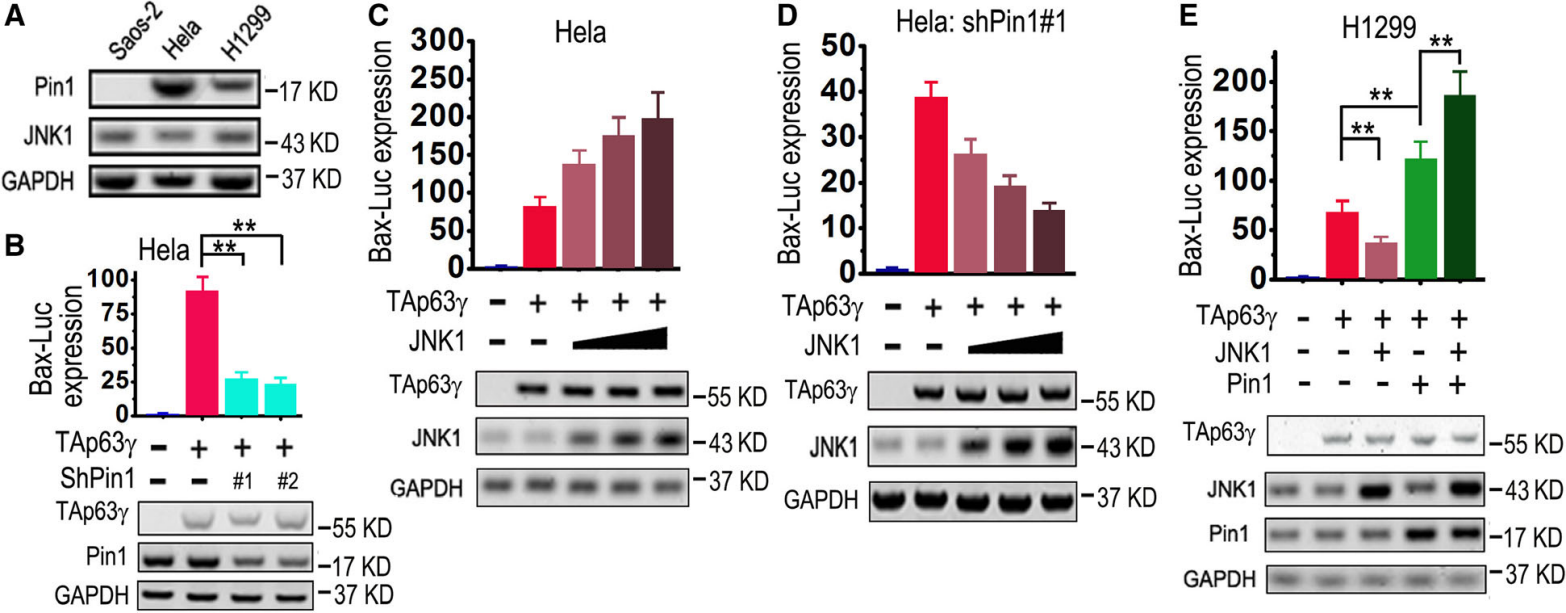
JNK1 may repress or promote transactivity of TAp63γ depending on Pin1 level. (A) Saos‐2, Hela and H1299 cells were lysed and indicated proteins were detected by means of IB analysis. (B) Hela cells were transfected with a mixture of Bax‐Luc and TK‐Renilla plus indicated plasmids. Bax‐Luc expression levels were measured and presented as mentioned above (*n* = 3), while IB analyses were performed to detect indicated proteins. Two‐tailed *t‐*test was used for comparison between two groups; **P < 0.01. (C, D) Hela cells, or Hela cells stably ablated with Pin1, were transfected with a mixture of Bax‐Luc and TK‐Renilla, plus HA‐TAp63γ and increasing amounts of JNK1 plasmid as indicated. Bax‐Luc expression levels were measured and presented as mentioned above (*n* = 3), while IB analyses were performed to detect indicated proteins. (E) H1299 cells were transfected with a mixture of Bax‐Luc and TK‐Renilla plus indicated plasmids. Bax‐Luc expression levels were measured and presented as mentioned above (*n* = 3), while IB analyses were performed to detect indicated proteins. Two‐tailed *t‐*test was used for comparison between two groups; **P < 0.01. The error bars (B–E) indicate SD.

As shown in Fig. 4A, there is a high level of endogenous Pin1 in Hela cells, while a moderate level in H1299. shRNA‐based knockdown of Pin1 can significantly impair TAp63γ‐mediated expression of Bax‐Luc in Hela cells (Fig. 4B). On the other hand, overexpression of JNK1 enhances TAp63γ‐mediated expression of Bax‐Luc in a dose‐dependent manner in Hela cells (Fig. 4C). This is contrary to our previous study in H1299 cells [27], as well as the results in Saos‐2 cells in the present study (Fig. 3). Intriguingly, in Hela cells ablated with Pin1, overexpression of JNK1 represses TAp63γ‐mediated expression of Bax‐Luc in a dose‐dependent manner (Fig. 4D). In H1299 cells, overexpression of Pin1 strikingly reverses effects of JNK1 on TAp63γ transactivity (Fig. 4E), just like it does in Saos‐2 cells (Fig. 3A).

These results suggest that JNK1‐mediated phosphorylation of TAp63γ at serine 12 can repress its transactivity, in the absence of abundant Pin1 (e.g., in Saos‐2 and H1299 cells, or Hela cells ablated with Pin1); in cells rich in Pin1 (e.g., Hela and Saos‐2 or H1299 ectopically overexpressing Pin1), the peptidyl‐prolyl isomerization of this phosphoserine‐proline (pS_12_‐P_13_) motif in the TAD of TAp63γ can strikingly activate its transcriptional activity (depicted as Graphical abstract figure).

## Discussion

The *p63* gene encodes multiple transcription factors [3]. Despite its low expression, TAp63γ plays key roles in quality control of germline cells, tumorigenesis, and aging, via its potent transactivity [7, 8, 9, 10, 11, 12, 13, 14]. We previously reported that Pin1 physically interacts with several isoforms of p63, including TAp63γ; Pin1 stabilizes TAp63α and ∆Np63α via mediating the isomerization of pT‐P‐P‐P‐P‐Y motif in the SAM and consequently impairing their affinity to E3 ligase WWP1 at this motif; however, the effect of this protein–protein interaction between TAp63γ and Pin1 was unknown [22]. In the present study, we find that Pin1 enhances transcriptional and pro‐apoptotic activities of TAp63γ (Fig. 1). On the other hand, we and others previously found that serine 12 (S**_12_**) in the TAD is critical to regulation of TAp63γ transactivity [24, 27]. S_12_ and the adjacent residue, proline 13 (P_13_), compose a potential Pin1‐binding site, which is supposed to lose the putative interaction by S12A mutation. We find that S12A mutant TAp63γ cannot be stimulated by Pin1 (Fig. 2A). Our further data show that this point mutation in TAp63γ significantly impairs its interaction with Pin1; the residual interaction between TAp63γ(S12A) and Pin1 indicates other binding sites of Pin1 than S_12_ in TAp63γ (Fig. 2B). Together, these results suggest that Pin1 promotes transactivity via binding to S_12_‐P_13_ in the TAD of TAp63γ. Since TAp63α and TAp63β also have this site, we speculate that Pin1 and JNK1 may regulate them in the same way. However, this regulation may not exist in △Np63 proteins, because they do not have the S_12_‐P_13_ motif in their truncated TAD [1].

S_12_ in TAp63γ is phosphorylated by IKKβ or JNK1, leading to an impairment of its transactivity [24, 27]. In our present study, we find that this inhibition of transactivity mediated by phosphorylation at this residue can be strikingly reversed by Pin1; in combination with JNK1, Pin1 can even enhance the transcriptional and pro‐apoptotic activities of TAp63γ to an extent that is higher than that in the absence of JNK1 (Fig. 3). JNK1 exhibits negative effects on TAp63γ activity in cells lacking abundant Pin1 proteins, while stimulates TAp63γ in cells rich in Pin1 (Fig. 4). Based on these results, we propose the following model to interpret the regulation of TAp63γ transactivity (as shown in Graphical abstract figure): TAp63γ with S_12_ unphosphorylated is moderately active; phosphorylation at this residue (pS_12_) mediated by IKKβ or JNK1 can repress its activity; in the presence of Pin1, isomerization of this pS_12_‐P_13_ motif makes TAp63γ hyperactive. Our data are helpful to elucidate the regulation of TAp63γ, which is an important transcription factor in tumorigenesis and germline quality control, as well as a potential therapeutic target against p53‐altered tumors [10, 16].

## Conflict of interest

The authors declare no conflict of interest.

## Author contributions

CL devised the hypothesis. XF, WH, KH, HC, LC, and SF designed and performed the experiments. W H and CL analyzed the data and wrote the manuscript.

## Data Availability

We state that our data will be available from the corresponding author upon reasonable request.
